# Supplementary material for: Dynamic and facilitated binding of topoisomerase accelerates topological relaxation
Source: Nucleic Acids Res. 2022 Apr 26;50(8):4659–68. doi: 10.1093/nar/gkac260 (PMC9071436; doi:10.1093/nar/gkac260)
Supplement: gkac260_Supplemental_Files [file gkac260_supplemental_files.zip › caption.docx]

Movie showing single examples of the stochastic trajectories simulated with the 2D random walk models presented in the main text. Model i corresponds to the static TopoII. Model ii corresponds to the randomly jumping TopoII. Models iii and iv correspond to TopoII that have biases to regions with large local density/curvature.
