# Supplementary material for: Dynamic and facilitated binding of topoisomerase accelerates topological relaxation
Source: Nucleic Acids Res. 2022 Apr 26;50(8):4659–68. doi: 10.1093/nar/gkac260 (PMC9071436; doi:10.1093/nar/gkac260)
Supplement: gkac260_Supplemental_Files [file gkac260_supplemental_files.zip › si_rev.pdf]

Dynamic and Facilitated Binding of Topoisomerase Accelerates Topological Relaxation: Supplementary Information

D. Michieletto,[1,2,*] Y. G. Fosado,[1,†] E. Melas,[1] M. Baiesi,[3,4] L. Tubiana,[5,6,7] and E. Orlandini[3,4]

[1]*School of Physics and Astronomy, University of Edinburgh, Peter Guthrie Tait Road, Edinburgh, EH9 3FD, UK*
[2]*MRC Human Genetics Unit, Institute of Genetics and Cancer, University of Edinburgh, Edinburgh EH4 2XU, UK*
[3]*Department of Physics and Astronomy, University of Padova, Via Marzolo 8, I-35131 Padova, Italy*
[4]*INFN, Sezione di Padova, Via Marzolo 8, I-35131 Padova, Italy*
[5]*Physics Department, University of Trento, via Sommarive, 14 I-38123 Trento, Italy*
[6]*INFN-TIFPA, Trento Institute for Fundamental Physics and Applications, I-38123 Trento, Italy*
[7]*Faculty of Physics, University of Vienna, Boltzmanngasse 5, 1090 Vienna, Austria*

MODEL AND METHODS

Unless otherwise stated, we model a torsionally relaxed (nicked) DNA plasmid about 3.6 kbp-long as a bead-spring polymer made of 500 beads (each bead having size $\sigma = 2.5$nm $= 7.3$ bp) connected in a ring. Inter-bead interactions are modelled with a purely steric truncated and shifted Lennard-Jones repulsion

$$U_{LJ}(r) = 4\epsilon\left[(\sigma/r)^{12} - (\sigma/r)^{6}\right] + \epsilon \quad (1)$$

for $r < r_c = 2^{1/6}\sigma$ and 0 otherwise. The TopoII-bound segment is modelled by allowing a 50 beads ($L = 360$ bp)-long segment to undergo strand-crossing with a small energy penalty ($A = 2k_BT$). This is done by modelling the interactions between this and all the other beads with a soft potential

$$U_s(r) = A(1 + \cos(\pi r/r_c)) \quad (2)$$

for $r < r_c = 2^{1/6}\sigma$ and 0 otherwise. Each bead is connected to its two neighbors along the ring by using a FENE potential

$$U_{FENE}(r) = -0.5KR_0^2 \log\left[1 - (r/R_0)^2\right] \quad (3)$$

with $K = 30\epsilon/\sigma^2$ and $R_0 = 1.6\sigma$. Finally, the persistence length of the DNA is modelled via Kratky-Porod potential

$$U(r) = \frac{k_BTl_p}{\sigma}(1 + \cos\theta) \quad (4)$$

where θ is the angle defined by two consecutive bond vectors along the polymer and $l_p = 20\sigma = 50$ nm is the persistence length of DNA.

We have chosen the length of the TopoII-bound segment for computational efficiency (longer segments lead to faster relaxations), but we have also checked that a more biologically realistic size of TopoII, i.e. 10 beads = 25 nm, gives the same qualitative results. The dynamical update of this "soft" segment is done by following one of the investigated models: either random jump, diffusion, jump to maximum local curvature and jump to maximum local density. For these different models of TopoII dynamics we study the relaxation of the knotting probability to its equilibrium value starting from a DNA molecule pre-knotted into a 5_1 torus knot (unless otherwise stated) and equilibrated at fixed topology (i.e. without TopoII) for at least 10^5 τ_B ($\tau_B = \xi\sigma^2/k_BT$ is the Brownian time, $\xi = 1$ the friction and $\simeq 10^5\tau_B$ is the longest relaxation time of the chain, see below). The molecular dynamics with implicit solvent (Langevin) simulations are evolved within the LAMMPS [1] engine coupled to custom-made C++ codes to perform the dynamic update of the TopoII region. (We provide sample codes in a github repository). Note that thanks to the coarse-grained nature of our simulations this model can also be applied to capture the behaviour of chromatin, simply by reducing the persistence length to about 100 nm [2]. The averages of the relaxation process are performed over at least 64, and up to 400, independent replicas. We follow the topology of the polymer by computing its Alexander determinant using the kymoknot software [3]. Since the knotting probability is computed over a binary value, the errors on the mean are obtained by the blocking method [4], i.e. by randomly assigning simulation replicas to different partitions, computing their mean knotting probability for each partition and finally computing the standard error of the mean (SEM) across partitions.

EQUILIBRATION

We start by analysing the behaviour of our system when we add a static TopoII. In particular we want to make sure that the system is in steady state before we add the crossable segment. A typical measure of the state of the polymer is the radius of gyration

$$R_g^2 = \frac{1}{2N^2}\sum_{i,j}^{N}[\boldsymbol{r}_i - \boldsymbol{r}_j]^2 \quad (5)$$

where $\boldsymbol{r}_i$ is the position of the bead i and N the length of the polymer. During the equilibration time R_g^2 settles

to a steady state value which is then perturbed when the static TopoII is introduced (Fig. S1). In particular one can see that by adding the crossable segment the size of the polymer increases, indicating the relaxation of the polymer to simpler topologies.

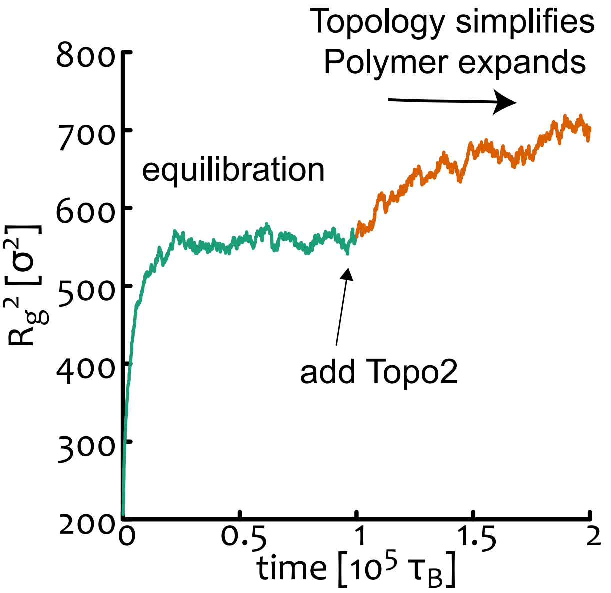

Figure S1. **Equilibration and relaxation. A.** Evolution of the squared radius of gyration R_g^2 in time during the equilibration step of a 5_1 knot (green) and then after the inclusion of a 50 beads-long Topo2 region (orange). R_g^2 increases because the topology is being simplified.

is about $0.4\,10^5\tau_B$. This time characterises the relaxation time of this polymer model. This should be compared with the typical inverse jumping rate at which enhanced topological simplification can be clearly noticed, i.e. about $1/k_j = 10^4\tau_B$ (see main text Figure 1B).

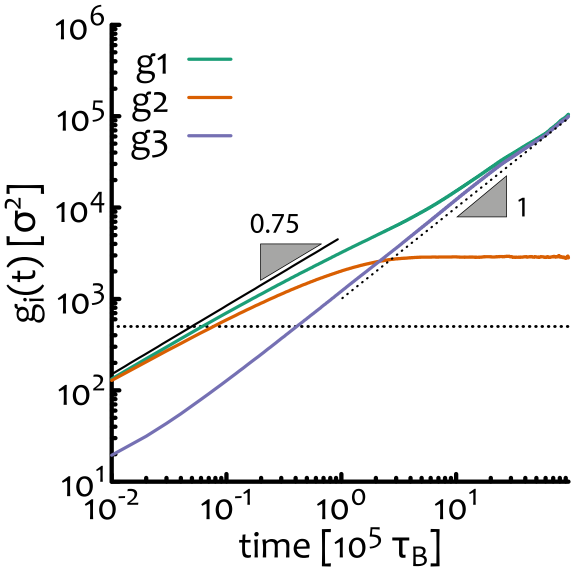

Figure S2. **Dynamics and conformational relaxation time.** This figure shows the mean squared displacements, g_i. Importantly, it shows the longest conformational relaxation time of the chain, i.e. when g_3 crosses the equilibrium $\langle R_g^2 \rangle$ (horizontal dotted line) is about $0.4\,10^5\tau_B$.

DYNAMICS AND RELAXATION TIME

In this section we show the dynamics of the $N = 500$, $l_p = 20\sigma$ DNA polymer. In Fig. S2 we report the mean squared displacement of the centre of mass,

$$g_3(t) = \langle [\boldsymbol{r}_{com}(t+t_0) - \boldsymbol{r}_{com}(t_0)]^2 \rangle \,, \quad (6)$$

of the monomers,

$$g_1(t) = \langle [\boldsymbol{r}(t+t_0) - \boldsymbol{r}(t_0)]^2 \rangle \,, \quad (7)$$

and that of the monomers in the frame of reference of the polymer centre of mass,

$$g_2(t) = \langle [\boldsymbol{r}(t+t_0) - \boldsymbol{r}_{com}(t+t_0) - \boldsymbol{r}(t_0) + \boldsymbol{r}_{com}(t_0)]^2 \rangle \,. \quad (8)$$

In each equation, the average is intended over t_0 or over t_0 and monomers (for g_1 and g_2). Importantly, one should notice that the longest relaxation time t_R, i.e. the time such that $g_3(t_R) \equiv R_g^2$ matches the size of the polymer,

STATIC TOPOII: COMPARING DIFFERENT NUMBER OF PARTITIONS

Here we address the question of how the unknotting rate changes when the TopoII region is either made of 1 long stretch of n_0 consecutive TopoII beads, or partitioned into m shorter segments n beads long such that they sum up to $n_0 = m \times n$. To this end we consider $N = 50$ TopoII beads randomly placed along the polymer as either $n = 50$,$m = 1$ (50×1), $n = 10$,$m = 5$ (10×5), $n = 5$,$m = 10$ (5×10) or $n = 1$,$m = 50$ (1×50). We also make sure that the different regions do not overlap to fix the same total number of crossable beads. These cases are compared in Fig. S3.

First, one should notice that the decay of the knotting probability $P_K(t)$ towards its equilibrium depends on m. Interestingly, it appears that there is an optimum for intermediate values of m while the case in which 50 crossable beads are randomly scattered along the polymer (but not overlapping) appears to be the slowest to simplify. At the same time, the large time equilibrium knotting probability is insensitive on the choice of m

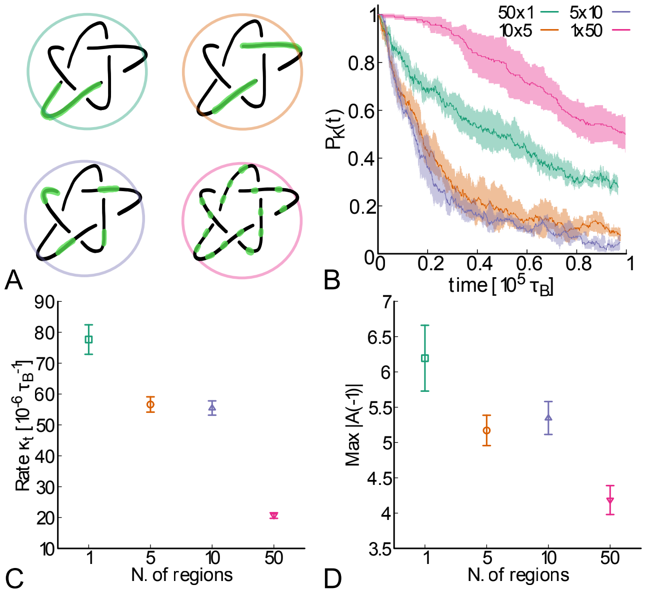

Figure S3. **Unknotting rate versus number of TopoII partitions. A.** Sketch of the set-up. The total number of Topo2 beads is kept constant at 50, while the partition (m) is varied: 50 consecutive beads (n=50, m=1, 50x1); 10 beads in 5 regions (n=10, m=5, 10x5), 5 beads in 10 regions (n=5, m=10, 5x10) and 1 bead in 50 regions (n=1, m=50, 1x50). **B.** Knotting probability as a function of time. Notice that the knotting probability decays with a rate that is non monotonic in m. **C.** Rate of strand-crossing events that also bring about a change in topology. This shows that the more the partitions the lower the rate at which strand-crossings happen. **D.** Maximum value of the Alexander determinant calculated at -1 and averaged across different replicas of the system. Notice that once again, this quantity is monotonically decreasing with m. Error bars and shaded areas represent the standard error of the mean.

(not shown). Further insight about this non-monotonic behaviour can be gained by looking at observables related to the topology of the polymer. As explained in the main text here we look at (i) the rate κ_t at which strand-crossing events generate a change in topology and (ii) the maximum value achieved by the Alexander determinant calculated at -1, $|A(-1)|$ (excluding the initial 5_1 topology). Notice that these two quantities are computed independently from the knotting probability $P_K(t)$. In particular one should not confuse κ_t with the decay rate of P_k. As shown in Fig. S3 this is in fact not the case; e.g. the 50x1 partition is the one with largest κ_t but it is not the fastest to simplify.

The interesting feature about Figs. S3C-D is that they both display a *monotonically* decreasing trend with the number of TopoII regions m while the decay rate of $P_K(t)$ is *non-monotonic* in m. This suggests that there is a competition between fast and accurate search in the space of topologies; e.g. while the 50x1 partition is the one with fastest sampling, it is also the one displaying more

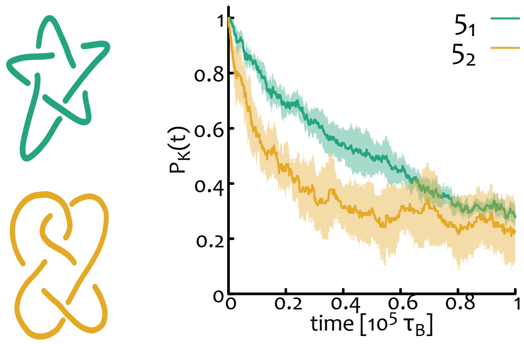

Figure S4. **Unknotting rate depends on initial knot.** A 5_2 knot displays a faster unknotting likely because it has unknotting number 1. Here we considered a static region.

complex topologies (large $Max|A(-1)|$). On the other hand, the partition 1x50 is the one with the least complex topologies explored but also the one with smallest κ_t. Interestingly, a balance between these two contributions appears to yield the most efficient and fastest topological simplification (largest decay rate of $P_K(t)$).

Intuitively, this can be explained as follows: increasing the number of partitions increase the probability that a TopoII partition is co-localised with the knotted portion of the knot. On the other hand, breaking up the partitions into too small segments hinders the efficiency of performing strand crossing moves, likely due to the presence of beads with excluded volume surrounding the short TopoII segments. In other words, while the single large partition ($m = 1$) is fast, it also meanders within regions of complex topology; at the same time the case with maximum number of partitions ($m = 50$) is the most precise at simplifying the topology but also the slowest to do so. Hence intermediate values of m bring about the most efficient strategy to simplify the topology of a given knot.

THE RELAXATION CURVES DEPEND ON THE INITIAL KNOT TOPOLOGY

In this section we test whether the topology of the initial knot affects the non-equilibrium simplification rate. To do this we compare the decay of the knotting probability of a system evolving from a twist 5_2 knot with our standard case starting from the torus knot 5_1 (see Fig. S4). We observe the former is simplified much faster than the 5_1 and we argue that this is due to the fact the 5_2 has unknotting number 1, i.e. a single judiciously chosen strand-crossing operation can turn the knot into an unknot, and this event has a non-zero chance to happen randomly within our simulation.

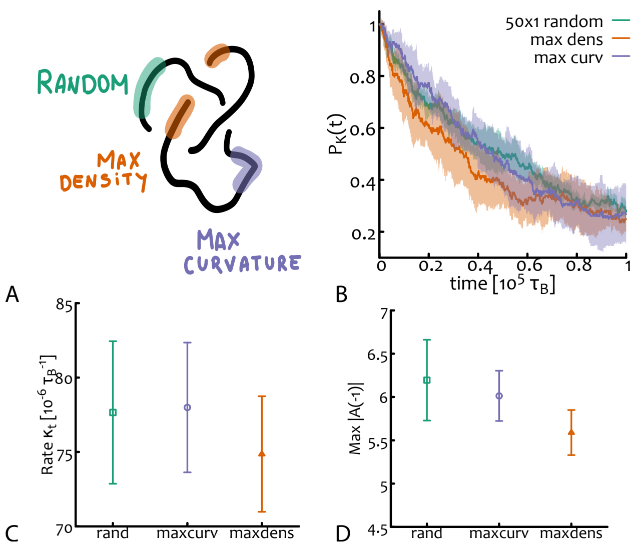

Figure S5. **Unknotting rate versus placement. A.** Sketch **B.** Comparison of the unknotting rate for different initial placement of a 50-beads spanning TopoII region. Random (rand); at location of maximum density (max dens) or maximum curvature (max curv). The maxdens strategy is the one yielding marginally faster simplification. **C.** Rate of topology changing moves. **D.** Maximum of the absolute value of the Alexander Determinant computed at -1. Notice that the error bars are SEM and the difference between data points is not significant.

TOPOII PLACEMENT

In this section we compare different initial placement of the TopoII region. We consider the same cases considered in the text, with the difference that this time the TopoII region cannot move. So it is the equilvalent of the static case, but starting from a judiciously chosen position which could be that of polymer maximum curvature or maximum density. These regions are computed as explained in the main text. As shown in Fig. S5, placing the TopoII region at the location of maximum local density yields a marginally faster unknotting rate, while the long term (equilibrium) unknotting probability is indistinguishable from the random case. Additionally, the calculation of the rate of topological change and the maximum value of topological complexity reveal only minimal differences between the different choices. Intriguingly, it appears that the choice to place TopoII in the region of maximum density brings about simpler topologies than the other cases; yet the difference between our data points is not statistically significant.

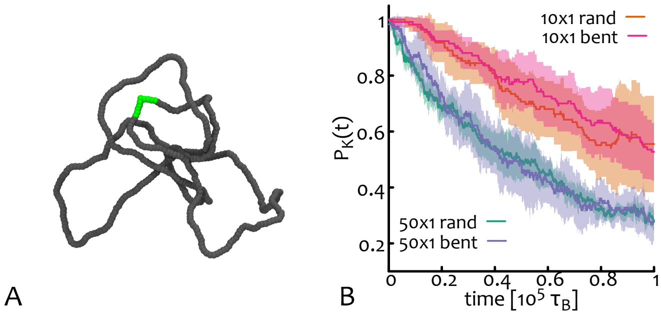

Figure S6. **Active bending of the TopoII segment does not accelerate unknotting. A.** Snapshot of the simulation. The bent TopoII segment is green. **B.** Knotting probability $P_K(t)$ as a function of time for bent and unbent (random) models, for two choices of TopoII region lengths (50 and 10 beads in a single region).

ACTIVE BENDING

In this section we ask whether a non-equilibrium process, i.e. that of actively bending the portion of the chain that is made crossable can speed up the topological simplification. With this aim, we introduce a harmonic bond between two beads placed 10 beads apart and symmetric with respect to the middle of the Topo2 region. The spring constant is set to $k_h = 20\epsilon/\sigma^2$ and the equilibrium length at $r_0 = 10\sqrt{2}$. Additionally, we make the triplet of beads in the middle of the Topo2 region fully flexible. With this choice, the equilibrium angle is $\theta = \pi/2$ in line with that observed in AFM [5, 6]. While one would expect that this model is enough to generate a bias towards unknotting, we find that, interestingly, this is not the case. In Fig. S6 we show that there is no significant difference between the knotting probability curves with bent and regular TopoII sections in the case of a static TopoII placed at random along the chain at time 0. We highlight that our results (albeit obtained with a very simplified model) align with current evidence suggesting that bending of DNA by TopoII is not a key determinant of knot simplifcation [5].

In the Figure we also compare the case in which the TopoII segments is made shorter (10 beads instead of 50). Again we don't see differences between the normal case and the one with bent segment. We also use this figure to show that the unknotting happens even with this shorter segment, but it takes longer to reach the same large-time behaviour.

RADIUS OF GYRATION FOR STATIC AND DYNAMIC MODELS

The steady state ensemble average radius of gyration for the different models take very similar values, suggesting that does not contribute to the different kinetics

of topological relaxation. In Fig. S7 we show R_g^2 after adding the Topo2 segment and for the same simulations that are reported in Fig. 4 of the main text. By comparing Fig. 4 in the main text and Fig. S7, one can notice that while the R_g has a similar kinetics across models, the topology has a very different kinetics.

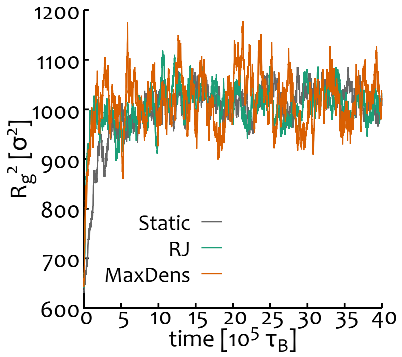

Figure S7. **Radius of gyration.** The radius of gyration for the polymers simulated with the different models are very similar. In the figure we show $\langle R_g^2(t)\rangle$ where the average is performed over 128 independent replicas for the static and random jump cases and 64 for the maximum density case. Here the polymer is N=1000 beads long and $l_p = 5\sigma$ as in Fig. 4 of the main text.

LARGE-TIME BEHAVIOUR

Here we discuss the large time, steady state behaviour of the models. The steady state knotting probability can be found as the the ratio of the times the polymer spends in a knotted state τ_K over the total time τ. For a purely diffusive model the knotting probability is $P_K^\infty = 1 - \tau_{0_1}/\tau = 1 - (\pi R_{0_1}^2/D)/(\sum_k \pi R_k^2/D) = \sum_{k>0_1} R_k^2/\sum_k R_k^2$, or the ratio between the sum of the volumes of the non-trivial topological spaces and the overall space. While purely diffusive or random jump processes are expected to yield the same P_K^∞, introducing either curvature or local density non-equilibrium jumps may yield different steady states. To test this we simulate long chains ($N = 1000, 2000$) with low persistence length, yielding $P_K^\infty \gtrsim 3\%$ [7]. In Fig. S8 we show that while P_K^∞ for the static TopoII model aligns with the one expected in equilibrium (a small model-dependent factor is expected), both dynamic models are characterised by broader distributions of P_K^∞ resulting into averages that are slightly larger than the static model. For large chains $N = 2000$ the relaxation from the 5_1 topology using the static Topo2 model is very slow and so we decided to also compute the steady state knotting probability starting from the unknot, 0_1. Given the finite time of our runs, these two cases represent the upper and lower bounds for the true steady state knotting probability. Importantly, irrespectively of the length of the polymer we see that the dynamic models yield a slightly broader distributions and larger average knotting probabilities.

DISTRIBUTION OF FIRST UNKNOTTING TIMES

It is interesting to note that in presence of crowding [8] or bounded domains [9], the mean passage time of a process is often an insufficient measure of the underlying process. In the case of the unknotting process, we can think our knotting probability curves to mirror a mean unknotting time. As shown in Fig. 2D of the main text, this mean time strongly depend on the binding kinetics and also on the search strategy. To further quantify this behaviour, we can measure he full distribution of first unknotting times, i.e. the time at which the substrate becomes unknotted for the first time in the simulation (it can then become knotted again). As shown, in Fig. S9, one can appreciate that for a static TopoII, the distribution is broad, covering the full length of the simulation. In stark contrast, the distributions become much narrower for the kinetically binding cases and in particular for the jump to max density (MD) strategy. For the random jump (RJ) we also observe some weak dependence of the distribution width on the jumping rate.

* davide.michieletto@ed.ac.uk
† yair.fosado@ed.ac.uk

[1] S. Plimpton, J. Comp. Phys. **117**, 1 (1995).
[2] C. A. Brackey, D. Marenduzzo, and N. Gilbert, Nature Methods **17**, 767 (2020).
[3] L. Tubiana, E. Orlandini, and C. Micheletti, Prog. Theor. Phys. Suppl. **191**, 192 (2011).
[4] M. Newman and G. Barkema, *Monte carlo methods in statistical physics chapter 1-4*, Vol. 24 (Oxford University Press: New York, USA, 1999).
[5] A. H. Hardin, S. K. Sarkar, Y. Seol, G. F. Liou, N. Osheroff, and K. C. Neuman, Nucleic Acids Research **39**, 5729 (2011).
[6] L. Alonso-Sarduy, C. Roduit, G. Dietler, and S. Kasas, FEBS Letters **585**, 3139 (2011).
[7] L. Coronel, E. Orlandini, and C. Micheletti, Soft Matter **13**, 4260 (2017).
[8] J.-H. Jeon, M. Javanainen, H. Martinez-Seara, R. Metzler, and I. Vattulainen, Phys. Rev. X **6**, 021006 (2016).
[9] T. G. Mattos, C. Mej, and R. Metzler, Phys. Rev. E **86**, 1 (2012).

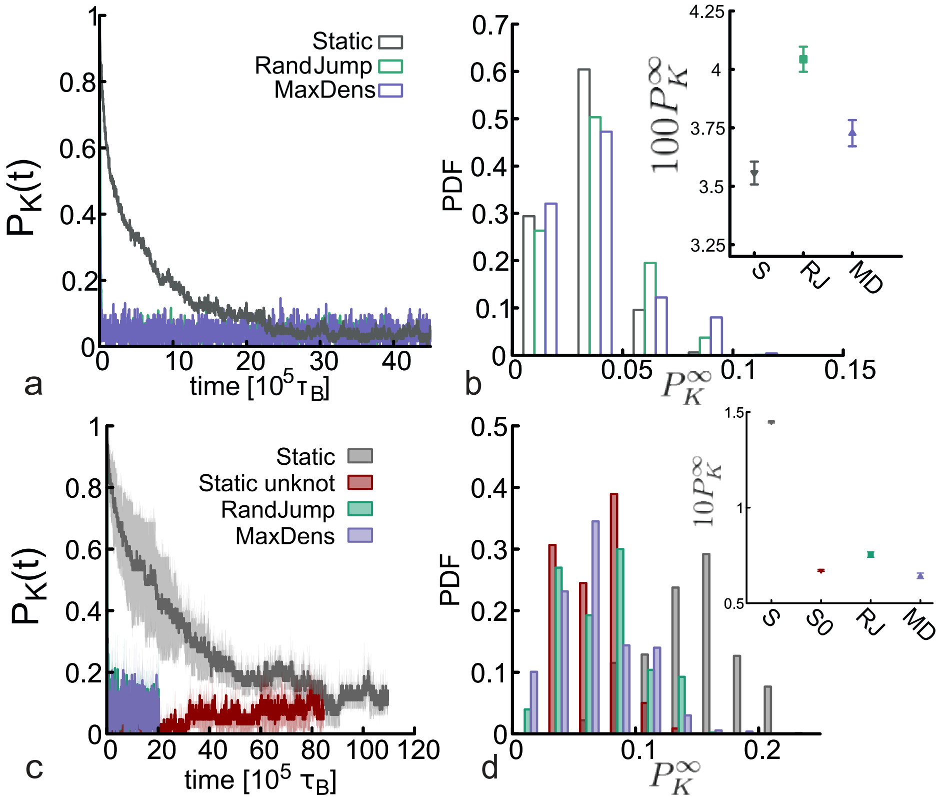

Figure S8. **Dynamic Binding Increases the Knotting Probability**. **a** Knotting probability curves starting from an ensemble of 5_1 knots. One can appreciate that the relaxation to steady state is about 1 order of magnitude faster in the case of dynamic TopoII. **b** The large-time steady state knotting probability (average of the curves in **a** after 30 $10^5\tau_B$) is lowest for the static case and aligning with the one expected in equilibrium (around 3% [7]). Introducing a dynamic TopoII slightly increases the steady state knotting probability. Error bars in inset represent standard error of the mean. In this panel, we consider a polymer $N = 1000$ beads long with $l_p = 5$ (a-b) and $N = 2000$ with $l_p = 5$ **c**. Knotting probability against time for a 5_1 knot $N = 2000$ beads long. In this case we also simulate the evolution starting from 0_1 which should converge to the same large time knotting probability. **d** Mean value of the knotting probability curves (average of the curves in **c** after 60 $10^5\tau_B$). One can appreciate that the large time knotting probability for the static case starting from the unknot is lower that both the dynamic RJ and MD cases, as seen for shorter chains in a-b.

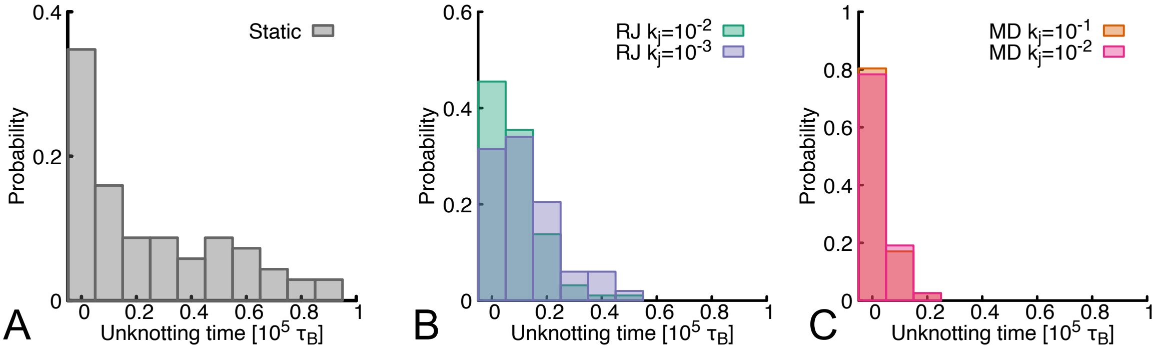

Figure S9. **Distributions of first unknotting times**. **A-C** Show the distributions of first unknotting times, i.e. the time at which the ring is first unknotted, during a simulation for the (A) static (B) random jump (RJ) and (C) binding to maximum density (MD) cases, respectively.
